# Localisation atypique de myomes en peropératoire: à propos de deux cas dont un dans un contexte d'urgence

**DOI:** 10.11604/pamj.2015.22.79.7846

**Published:** 2015-10-01

**Authors:** Boureima Kinda, Charlemangne Ouédraogo, Edgar Ouagré, Nadine Ghilat, André Simporé, Papougnézambo Bonkougou, Joachim Sanou

**Affiliations:** 1 Département d'Anesthésie et Réanimation, CHU Yalgado Ouédraogo, Ouagadougou; 2 Département de Gynécologie et Obstétrique, CHU Yalgado Ouédraogo, Ouagadougou; 3 Département de Chirurgie et Spécialités Chirurgicales, CHU Yalgado Ouédraogo, Ouagadougou; 4 Service de Gynécologie et Obstétrique du Centre Médical de Saint Camille, Ouagadougou

**Keywords:** Myomes, intestin, paroi abdominale, utérus, myomas, intestine, abdominal wall, uterus

## Abstract

Les fibromyomes sont des tumeurs bénignes de localisation utérine courante. Elles sont fréquentes chez la femme noire, le diagnostique est échographique et anatomo-histologique. La localisation extra utérine est rare et de physio-pathogénie mal connue. Cette localisation pose des problèmes de diagnostique. Nous rapportons deux cas cliniques de fibromes localisés sur le segment sigmoïdien de l'intestin et sur la paroi interne du muscle transverse de l'abdomen d'une part chez une patiente âgée de 41 ans et d'autre part en région épigastrique, chez une patiente de 47 ans. Toutes les patientes ont été opérées respectivement l'une de myomes utérins pour infertilité et l'autre de laparotomie en urgence pour syndrome sub-occlusif au cinquante huitième jour post hystérectomie. Ces observations doivent inciter les cliniciens ou les radiologistes à réaliser un bilan étendu à la recherche de localisation extra-utérine avant une myomectomie ou hystérectomie car un myome peut en cacher un autre en dehors de l'utérus.

## Introduction

Les myomes sont des tumeurs bénignes, typiques à l'utérus, très fréquentes chez la femme noire, responsables d'infertilité et de métrorragie [1]. Leurs localisations extra-utérines sont rares mais posent de nombreux problèmes, notamment de diagnostique préopératoire, de prise en charge per-opératoire et post opératoire, d’évolution et de pronostic [2]. Nous rapportons deux cas cliniques, dont un dans un contexte d'urgence, où trois localisations extra-utérines différentes ont été identifiées. Nous avons discuté des mécanismes à l'origine de l'implantation et du développement extra-utérins de ces lésions.

## Patient et observation

**Figure 1:**
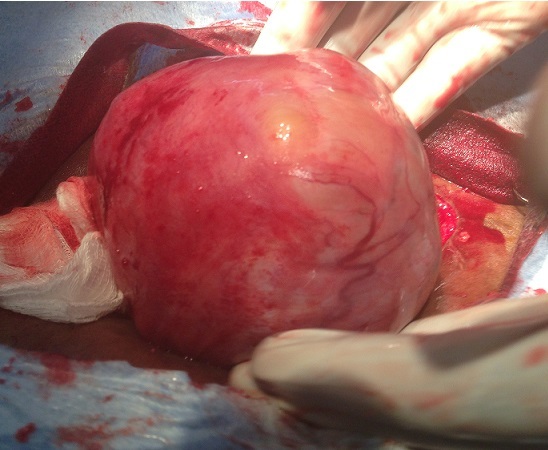
Myome épigastrique

**Observation 1:** Mm K K, 47ans, G1 P1, a été admise en urgence pour un syndrome sub-occlusif survenu au cinquante huitième jour en post-opératoire d'une hystérectomie. La visite pré-anesthésique réalisée au lit de la patiente en urgence la veille de l'intervention note une patiente ASA II (American Society of Anaesthesia II), dyspnéique, anxieuse, fébrile à 38°5 C. On notait des vomissements sans arrêt de matière et de gaz. La tension artérielle était de 120 mmHg pour la systole et 70 mmHg pour la diastole. Une masse de consistance dure, flottante, sensible, était visible et palpable en région épigastrique. L’échographie décrivait une masse tissulaire de 113 mm x 89 mm, sus ombilicale, vascularisée, évoquant un myome ou un processus expansif. Une sonde gastrique et une réhydratation hydro-électrolytique ont été initiées pendant 24 heures, puis une laparotomie exploratrice sous anesthésie générale en urgence a été indiquée et a permis d'extraire une volumineuse tumeur (Figure 1) dont les résultats anato-pathologiques sont: sur le plan macroscopique le prélèvement mesure 120x100x80 mm et pèse 643 g, l'aspect est fibreux, fasciculé; sur le plan microscopique, on note une prolifération de léiomyocytes d'architecture fasciculée sans atypies cytonucléaires, entremêlés de fibroblastes et de bandes de collagènes, des vaisseaux à paroi propres. La conclusion étant celle de fibromyomes dans les deux prélèvements.

**Figure 2:**
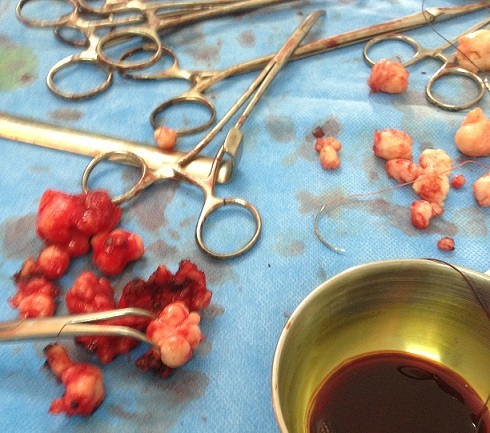
Noyaux utérins (en haut) sigmoidiens et pariétaux (en bas)

**Observation 2:** Mm N K, 41 ans, nullipare, admise au bloc pour myomectomie pour la deuxième fois en 3 ans d'intervalle pour récidive de fibromes utérins, responsable d'infertilité primaire, de douleurs pelviennes et de métrorragie. L’évaluation pré anesthésique réalisée une semaine avant l'intervention avait conclue à une patiente ASA 1, sans autre particularité clinique. L’échographie décrivait un utérus poly-myomateux. Une rachianesthésie a été préconisée. Une myomectomie avant procréation médicalement assistée a été décidée devant le refus d'une hystérectomie par la patiente. En peropératoire, des noyaux sont extraits d'une part de l'utérus, et d'autre part du sigmoïde intestinal et de la paroi interne du muscle transverse de l'abdomen ainsi que 350 mL de pertes sanguines compensées par un litre de ringer lactate. Les résultats histo-diagnostiques sont les suivants: 18 noyaux myomateux pesant ensemble 90 g, d'aspect blanchâtre, ferme, fasciculé et fibreux, prolifération de léiomyocytes sans atypie cyto-nucléaires, fibroblaste et collagène, observés pour le premier prélèvement (Figure 2). Pour le second prélèvement (Figure 2), sept fragments nodulaires comportant chacun de petites formations nodulaires secondaires, l'ensemble pesant 150 g, l'aspect est blanchâtre, fibreux, fibromatose profonde de type desmoide. La conclusion étant celle de fibromyomes dans les deux prélèvements.

## Discussion

Le myome utérin ou léiomyome est une tumeur bénigne du tissu musculaire lisse de l'utérus [2]. Il est aussi désigné sous le terme de fibromyome ou fibrome (lésion conjonctive bénigne) par abus de langage. Dans nos deux cas l'histo-diagnostique a conclu à une léomyomatose et une fibrose. Près de 50% des femmes noires après 30 ans portent des fibromes [2]. Les complications (hémorragie, infertilité, complications obstétricales, douleurs) sont rares [3]. L’échographie est l′examen diagnostique de référence des fibromes et permet une cartographie des lésions. La localisation extra-utérine est rare, voire controversée [4]. Dans notre série la tumeur extra-utérine de Mm K K, a été décelée en préopératoire. Un fibrome ou bien un processus expansif avaient été évoqués. Le fibrome a été confirmé par les résultats de l'anatomopathologie. Les processus expansifs sont essentiellement les léimyosarcomes, les adénomyomes qui sont plutôt des lésions invasives. Les localisations extra-utérines de myomes rapportées dans la littérature sont les léiomyomes métastatiques bénins, les léimyomatoses péritonéales disséminées, les léiomyomatoses intraveineuse, et les léiomyomes rétro-péritonéaux. Roue et al [4] ont décrit trois cas de léimyomes extra-utérins au niveau du ligament rond, du ligament large et de l'ovaire. Ziouziou et al [5]. rapporte un cas de léimyome rétro-péritonial (LRP), tandis Poliquin et al [6]. dans une revue de littérature rapporte 105 cas de LRP entre 1941 et 2007.

**Figure 3:**
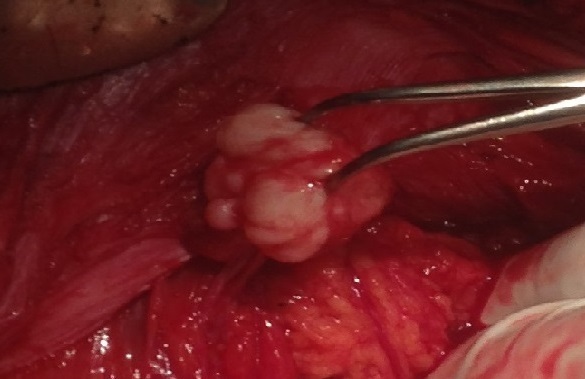
Fibrome fixé sur la paroi abdominale interne (en cours d"extraction)

Dans notre série nous avons éliminé un adénocarcinome péritonéal et colique et un sarcome de la paroi abdominale qui sont plutôt des tumeurs malignes [7]. Nous avons écarté également une transformation de lésions endométriosiques extra-utérines chez Mme KK, au vue des résultats microscopiques de l'anatomopathologie. Pour l'origine de ces fibromes nous évoquons trois hypothèses: premièrement des noyaux d'origine utérine, adhérenciels aux organes de voisinage, ont été ignorés pendant la myomectomie antérieure ou l'hystérectomie précédente puis se sont développés secondairement à leur propre compte. Deuxième hypothèse: des noyaux sous muqueux ou séreux ont été libérés de l'utérus lors de la première intervention sans avoir été extirpés hors de l'abdomen, puis se sont développés à leur propre compte grâce à une néo vascularisation au contact du sigmoïde ou de la paroi abdominale. Enfin, dernière hypothèse, à partir des organes ou elles ont été diagnostiquées en peropératoire des lésions primaires se sont développées in situ mais n'avaient pas été recherchées en préopératoire. Nous retenons la première hypothèse. En effet selon Christin-Maitre et al., les myomes sous-muqueux et sous-séreux peuvent se détacher exceptionnellement du myomètre et se développer grâce à leur accolement aux tissus voisins. Dans notre étude, nous avons une forte présomption qu'il pourrait s'agir de noyaux utérins pédiculés adhérés aux organes de voisinages et ignorées pendant la chirurgie, puis se sont développées grâce à une néo vascularisation. En effet Kho et al [8]. dans leur série avait constaté que 67% des localisations extra-utérines avaient des antécédents chirurgicaux de myomectomie tandis que Poliquin et al constatent que 40% de localisations rétro-péritonéales avaient des antécédents d'hystérectomie. Mais nous n’écartons pas la possibilité de lésion primaire. Aucun de ces arguments ne peut être réfuté catégoriquement car ces lésions ont été visualisées en peropératoire (Figure 3) et confirmées en post-opératoire, par les résultats anatomopathologiques qui sont en faveur de fibromyomes. On retient de ces 2 cas que des fibromyomes peuvent en cacher d'autres situés sur l'utérus ou hors de l'utérus. En l'absence d'un diagnostic pré-préopératoire (**Observation 1**), une hystérectomie ne résout pas le problème de ces localisations extra-utérines. Le bilan clinique pré-opératoire doit songer à d'autres localisations.

## Conclusion

Nous avons relevé trois localisations extra-utérines de fibromes en per et postopératoire. Nous avons discuté de l'origine de ces trois lésions qui ont pour point commun les antécédents de chirurgie de l'utérus pour myomes. Un recueil de revue de littérature actualisé devrait permettre de décrire l'incidence de ces tumeurs afin de lever la controverse sur leur existence, car elles sont de plus en plus révélées dans la littérature.
